# Prevalence and effect of *Plasmodium* spp. and hookworm co-infection on malaria parasite density and haemoglobin level: a meta-analysis

**DOI:** 10.1038/s41598-022-10569-2

**Published:** 2022-04-27

**Authors:** Aongart Mahittikorn, Frederick Ramirez Masangkay, Giovanni De Jesus Milanez, Saruda Kuraeiad, Manas Kotepui

**Affiliations:** 1 Department of Protozoology, Faculty of Tropical Medicine, Mahidol University, Bangkok, Thailand; 2 Department of Medical Technology, Faculty of Pharmacy, University of Santo Tomas, Manila, Philippines; 3 Medical Technology, School of Allied Health Sciences, Walailak University, Tha Sala, Nakhon Si Thammarat, Thailand

**Keywords:** Malaria, Parasitic infection

## Abstract

The dual effects of co-infection of *Plasmodium* spp. and hookworm on malaria remain under debate. This study investigated prevalence, prevalence odds ratio (POR) of co-infection and impact of co-infection on malaria parasite density and haemoglobin levels in comparison to *Plasmodium* mono-infection. The protocol for this systematic review and meta-analysis is registered at PROPERO under ID: CRD42020202156. Relevant literatures were obtained from PubMed, ISI Web of Science, and Scopus on 25 December 2020. Mean difference (MD) and confidence interval (CI) of malaria parasite density and haemoglobin were compared using a random effect model. Heterogeneity was assessed using Cochrane Q and I^2^ statistics. Publication bias was determined by visualising funnel plot asymmetry. Of 1756 articles examined, 22,191 malaria cases across 37 studies included 6096 cases of co-infection of *Plasmodium* spp. and hookworm. The pooled prevalence was 20% (95% CI 15–26%, I^2^ 99.6%, 37 studies) and was varied in terms of geographical region. Co-infection occurred by chance (OR 0.97, *p* 0.97, 95% CI 0.73–1.27, I^2^ 95%, 30 studies). The mean malaria parasite density for co-infection (478 cases) was similar to *Plasmodium* mono-infection (920 cases) (*p* 0.24, MD 0.86, 95% CI − 0.58–2.29, I^2^ 100%, 7 studies). The mean haemoglobin level for co-infection (90 cases) was similar to *Plasmodium* mono-infection (415 cases) (*p* 0.15, MD − 0.63, 95% CI − 1.49–0.23, I^2^ 98%, 4 studies). Co-infection was common and occurred by chance but varied by geographic region. Further studies are required to investigate the mechanism of hookworm infection on malaria severity. Additionally, detection of hookworm infections among patients with malaria in endemic areas of both diseases is recommended to prevent severe malaria.

## Introduction

The most common soil-transmitted helminths (STHs) that cause human intestinal helminthiasis are *Ascaris lumbricoides*, *Trichuris trichiura* and hookworms (*Necator americanus* and *Ancylostoma duodenale*)^1^. STHs are common in areas where the capability to provide basic healthcare is limited, such as Sub-Saharan Africa (SSA)^2–5^. However, malaria remains the most devastating health problem in the SSA, causing an estimated 40,000 deaths each year^6^. In the SSA, the main cause of death of children younger than five is malaria induced by *Plasmodium falciparum*^7^. As malaria and STHs overlap in geographical distribution, co-infection of *Plasmodium* spp. and hookworm in the same individuals has frequently occurred. Various studies have demonstrated the impact of *Plasmodium* spp. and hookworm infection on the severity of malaria, including parasite density and severe anaemia^8–13^, demonstrating that the mean haemoglobin concentration is lower in patients with co-infection than those with either hookworm or malaria infection alone^8,9^. Additionally, the prevalence odds ratio (POR) of anaemia was higher in patients with co-infection than those with either hookworm or malaria infection alone^10^. A previous study demonstrated that a higher prevalence of only hookworm infection among patients with malaria and most of the coinfected patients had moderate (25%) or heavy (13%) hookworm infections^11^. Another study showed a positive interaction between hookworm infections with *Plasmodium* densities^12^ and that patients with heavy hookworm infection had a higher malaria parasite density than those with low or moderate hookworm infection^12,13^. A previous study suggested that the intensity of hookworm infection steadily increased with age, which indicated that hookworms might suppress host immune responses^14^. Nevertheless, according to one report, no association between hookworm infection and clinical malaria was found, even with heavy hookworm infection^15^. Various studies compared the magnitude of *Plasmodium* spp. and STHs’ co-infection between malaria and STHs^16–19^. A previous study found that although *p. falciparum* infection could induce a higher level of proinflammatory markers than those with *S. haematobium* infection, no effect of *S. haematobium* was found on patients with *P. falciparum* gametocyte carriage^16^. Another study reported that *S. haematobium* infection could induce immunity against malaria by regulation of proinflammatory cytokines, such as IL-10 and IFN-γ production^18^. Interestingly, a previous study found that *S. haematobium* infection could increase the risk of *Plasmodium* infection in children if *Trichuris trichiura* or hookworm infection was the concurrent infection^20^. This indicates that multiple infections of STH could affect malaria. Furthermore, a previous study suggested that helminth infections can stimulate type 2 immune responses, which are important for the induction and development of humoral immune responses for controlling malaria parasites in the blood and protecting the patients against severe malaria^21^.

As the occurrence of co-infection will influence the planning of integrated intervention strategies for malaria and hookworm, data on *Plasmodium* spp. and hookworm infection is crucial for developing integrated control efforts for disease elimination. Although various studies aimed to assess the magnitude of *Plasmodium* spp. and STH co-infection, there are no adequate reports that explain the correlation of *Plasmodium* spp. and hookworm co-infection with the level of malaria parasitaemia and haemoglobin level.

## Objectives

This study aimed to determine the effect of hookworm infection on *Plasmodium* parasitaemia and haemoglobin levels using the meta-analysis approach.

## Methods

### Protocol and registration

The present systematic review and meta-analysis followed the Preferred Reporting Items for Systematic Reviews and Meta-Analyses (PRISMA) (Checklist S1). The protocol was registered at the International Prospective Register of Systematic Reviews (PROSPERO) with registration ID: CRD42020202156.

### Definitions of co-infections

Co-infections were defined as these criteria: (a) presence of both parasites in the study subjects; (b) presence of helminths in patients with malaria; or (c) presence of *Plasmodium* in patients with helminths infection.

### Literature search

Searches for relevant articles were performed in PubMed, Web of Science and Scopus. The following combination of keywords were checked for a Medical Subject Heading term: ‘(malaria OR Plasmodium) AND (hookworm OR Ancylostoma OR Necator)’, as shown in Supplementary Table S1.

### Eligibility criteria

The inclusion criteria were prevalence studies (descriptive cross-sectional) that met the definition of co-infection. Case–control studies were excluded because their reported prevalence of co-infection could not be pooled for meta-analysis. The following studies were also excluded: review articles, in vitro or in vivo studies, intervention studies and randomised control trials. Other exclusion criteria were articles written in non-English language, studies without the full-text available, studies for which the data could not be extracted, books or book chapter, studies on the same group of participants, case reports or case series, protocol and questionnaires.

### Study selection and data extraction

Two independent authors (AM, MK) selected the potentially relevant studies based on eligibility criteria. Any discrepancy between the two reviewers was resolved by discussion or request of the second author (FM) for the conclusion. Data extraction was performed by the same authors. The following data were extracted for the pilot standardised datasheet: the name of the first author, publication year, study location, year that the study was conducted, study design, characteristics and number of participants, number of malaria cases, number of hookworm cases, number of co-infection, number of single infections, malaria parasite density and haemoglobin level.

### Quality of included studies

The quality of the included studies was assessed using the Newcastle–Ottawa Scale (NOS) for assessing the quality of nonrandomised studies in meta-analyses (Supplementary Table S2). NOS provided a star system for judging the included studies based on the selected study groups, comparability of the groups and ascertainment of either exposure or outcome of interest^22^. The comparability criteria that are not applicable to most study types were defined as “not applicable” since no data from control groups were unavailable. For this study, any included study rated more than 6 out of 7 stars indicated a high-quality study. Any included study rated 4–5 stars indicated moderate quality, whereas any study rated below 3 stars indicated poor quality.

### Outcomes

The outcomes of the study were (i) magnitudes of co-infection, (ii) magnitude of parasitaemia, (iii) magnitude of malaria severity (anaemia).

### Statistical analysis

The pooled prevalence of *Plasmodium* spp. and hookworm co-infection was estimated by the random effect model using the number of patients with co-infection and the total number of patients with malarial infection. The pooled prevalence odds ratio (POR) of hookworm infection in patients with malaria and in patients without malaria were estimated by the random effect model based on the number of patients infected with hookworm per all malaria cases, and the number of patients with hookworm per non-malaria cases. The mean parasite density and haemoglobin level among patients with co-infection and patients with *Plasmodium* spp. mono-infection were compared and shown as the weight mean difference with a 95% confidence interval (CI). If the included studies reported the median and interquartile range of parasite density or haemoglobin level, the mean and standard deviation were calculated by a formulation published elsewhere^23^. A statistical model for pooling the data was the random effect model in the case of substantial heterogeneity (I^2^ > 50% or Cochrane Q < 0.05) across the included studies and the fixed effect model in the case that heterogeneity across the included studies was not substantial (I^2^ < 50% or Cochrane Q > 0.05). Meta-regression analysis was performed to identify the source(s) of heterogeneity of the outcomes.

### Publication bias

Publication bias among the included studies was assessed through visualisation of the funnel plot asymmetry and Egger’s test. The funnel plot demonstrated the effect size and standard error of the effect size. The significance of Egger's test (p < 0.05) demonstrated the asymmetrical distribution of the funnel plot and suggested that publication bias was caused by the small-study size.

## Results

### Search results

**Figure 1:**
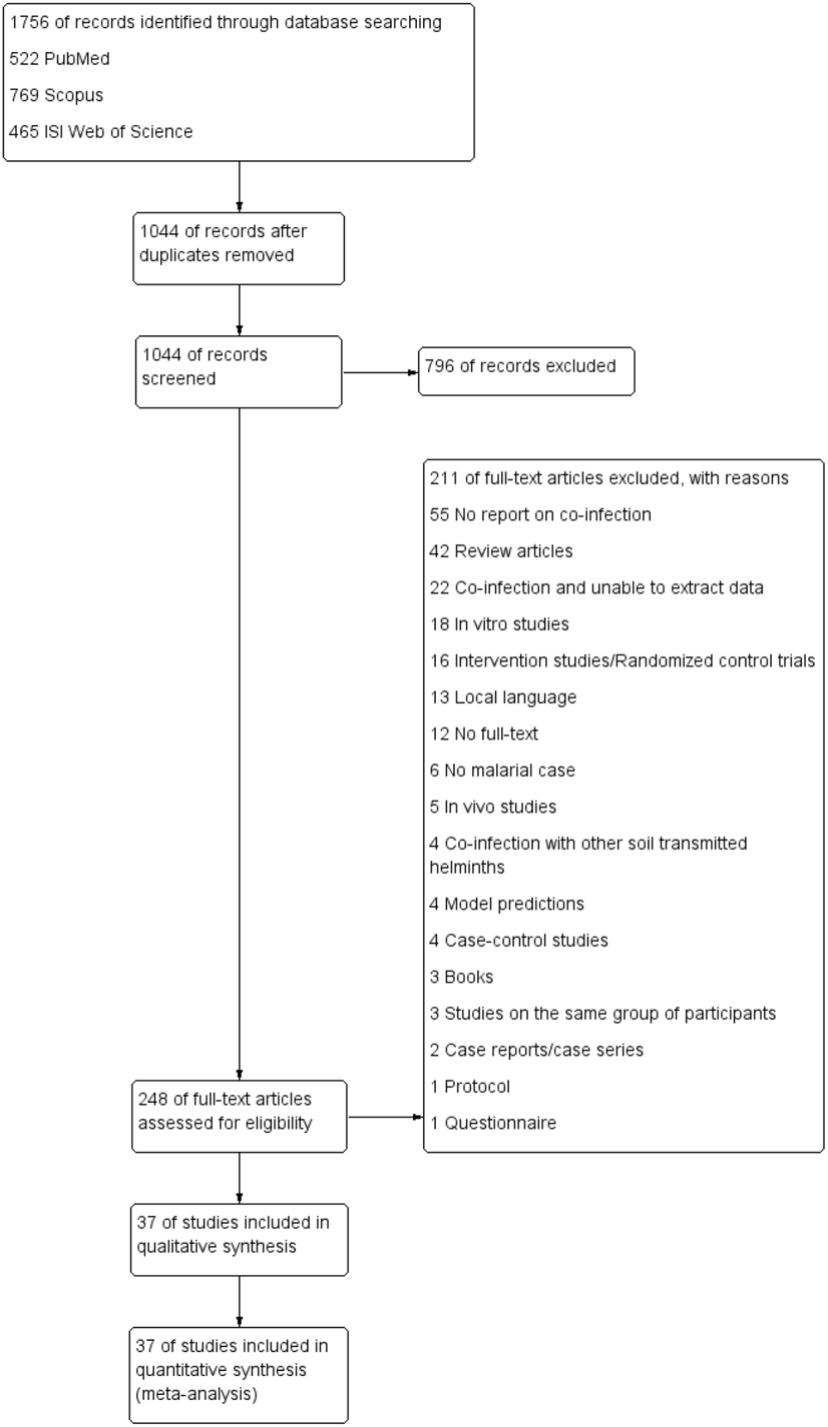
Study flow diagram.

The searches retrieved 522, 769 and 465 articles from PubMed, Scopus and ISI Web of Science, respectively. Of the 1756 studies screened, 712 were duplicates and were removed. Of the 1044 articles screened for titles and abstracts, 796 articles were excluded due to irrelevant articles. Of the 248 articles examined for full texts, 211 articles were excluded for the following reasons: 55 showed no report on co-infection, 42 were review articles, 22 showed co-infection but the data could not be extracted, 18 were in vitro, 16 were intervention studies/randomised control trials, 13 were published in local languages, 12 were not full-text, 6 were not malarial case, 5 were in vivo, 4 were co-infection with other nematodes, 4 were model prediction, 4 were case–control studies, 3 were books, 3 were studies on the same group of participants, 2 were case reports/case series, 1 was protocol and 1 was a questionnaire. Finally, 37 studies met the inclusion criteria and were included in the analysis (Fig. 1).

### Characteristics of the included studies

Characteristics of the included studies are shown in Table 1. Most of the included studies were cross-sectional studies (35/37, 94.6%). Most of the included studies reported *Plasmodium* spp. and hookworm co-infection in Africa (34/37, 91.9%)^8–13,20,24–50^ while the remaining studies were from Asia (1 Thai-Burmese border, 1 Indonesia)^51,52^ or South America (Brazil)^53^. Most of the included studies conducted in Africa were in Nigeria (8/34, 23.5%)^11,24,28,32,33,45,46,50^, followed by Ethiopia (6/34, 17.6%)^8,9,12,13,31,42^, Tanzania (4/34, 11.8%)^38–40,49^, Uganda (4/34, 11.8%)^34,36,44,47^, Coˆted’Ivoire (3/34, 8.8%)^10,41,48^, Ghana (3/34, 8.8%)^26,27,35^, Kenya (2/34, 5.88%)^30,37^, Gabon (2/34, 5.88%)^20,25^ and Cameroon. One study covered three countries, including Kenya, Ethiopia and Uganda^29^.

**Table 1:** Characteristics of the included studies.

No.	Author, year	Study area (years of the survey)	Study design	Participants, malaria cases	Co-infection with hookworm				Malaria mono-infection				Hookworm mono-infection/All infections (n/N, %)	No *Plasmodium* or hookworm infection (n)
					*Plasmodium* spp. (n), mean parasite density (per μL)	Hb (g/dL)	Age/male (n, %)	Anemia (n, %)	*Plasmodium* spp. (n), mean parasite density (per μL)	Hb (g/dL)	Age/male (n, %)	Anemia (n, %)		Hb (g/dL)
1	Adedoja et al., 2015	Nigeria (2012–2013)	Cross-sectional study	1017 primary school pupils, 355 *P. falciparum*	*P. falciparum* (61), 350/μL		4–9 (25/446), 10–15 (36/572)/male 35/519, female 26/498		*P. falciparum* (209), 270/μL		4–9 (101/445), 10–15 (108/572)/male 104/519, female 105/498		229/486 (47.1)	
2	Adegnik et al., 2010	Gabon (2003–2004)	Cross-sectional study	388 pregnant women, 98 *P. falciparum*	*P. falciparum* (10)				*P. falciparum* (35)				34 (including co-infections)	
3	Adu-Gyasi et al., 2018	Ghana (2015–2016)	Cross-sectional study	1826 residents, 441/1,569	78/1094				311				90/290 (31%)	
4	Amoani et al., 2019	Ghana	Cross-sectional study	984 community members, 122 *P. falciparum*	*P. falciparum* (63)				*P. falciparum* (59)				40/103 (38.8%)	
5	Babamale et al., 2016	Nigeria (2015)	Hospital-based study cross-sectional study	300 pregnant women, 90 *P. falciparum*	*P. falciparum* (11), 859.67 ± 53.09 (11)				*P. falciparum* (52), 1034.90 ± 234.53 (14)				NA	
6	Babamale et al., 2018	Nigeria (2015)	Cross-sectional study	508 people in communities, 300 *P. falciparum*	*P. falciparum* (142), 1836.5 ± 2028.9 Light infection 2007.54 ± 2079.016 (26), Moderate infection 1872.78 ± 2107.342 (77), heavy infection 1650.90 ± 1868.288 (39) Average 1843.74 ± 2018.2 (142)				*P. falciparum* without hookworm (158), 2145.88 ± 2577.542 (158)				142 (including co-infections)	
7	Boel et al., 2010	Thai-Burmese border (1996 and 2007)	Cross-sectional study	829 pregnant women, 153/796 (53 *P. falciparum*, 83 *P. vivax*, 17 mixed infection)	57 (19 *P. falciparum*, 29 *P. vivax*)				44 (15 *P. falciparum*, 25 *P. vivax*)				141/355 (39.7%)	
8	Brooker et al., 2012	Kenya (2008–2009), Ethiopia (2008–2009), Uganda (2006 and 2009)	Cross-sectional study	28,050 school aged children, 2974 *P. falciparum* (estimated from prevalence) Kenya (1,144), Ethiopia (7), Uganda 2006 (582), Uganda 2009 (1,241)	*P. falciparum* (529), Kenya (161)*,* Ethiopia (3), Uganda 2006 (118) *P. falciparum*, Uganda 2009 (247)				2445 *P. falciparum*				3,848 (including co-infections), Kenya (2,091), Ethiopia (1,156), Uganda 2006 (346), Uganda 2009 (327)	
9	Burdam et al., 2016	Indonesia (2013)	Cross-sectional study	629 children aged 1 to 59 months, 72 malaria cases (47/533 *P. vivax*, 21 *P. falciparum*, 1 *P. malariae*, 3 mixed infection)	6				66				13/34 (38.2%)	
10	Bustinduy et al., 2013	Kenya (2009–2010)	Cross-sectional study	2030 children, 333 *P. falciparum*	95 *P. falciparum*				238 *P. falciparum*, 98 Pure *P. falciparum*				406 (including co-infections)	
11	Degarege et al., 2009	Ethiopia (2007)	Cross-sectional study	1802 acute febrile patients, 458 (366 *P. vivax*, 72 *P. falciparum*)	173, light hookworm (146) 6510; moderate hookworm (21) 9920; heavy hookworm (7) 18,256 11/247 severe malaria, 236/247 uncomplicated malaria		< 5 (12), 5–14 (37), ≥ 15 (124) 80/173		Malaria without hookworm 26/203 severe malaria, 177/203 uncomplicated malaria				NA	
12	Degarege et al., 2012	Ethiopia (2010–2011)	Cross-sectional study	1065 febrile patients, 306 malaria cases, 138 *P. falciparum*, 154 *P. vivax,* 14 mixed infection	7 (1 *P. falciparum*, 6 *P. vivax*)				86				18/173 (10.4%)	
13	Dejon-AgobeÂ et al., 2018	Gabon (2012–2014)	Prospective longitudinal study	754 children, 167 *P. falciparum*	11 *P. falciparum*				115 *P. falciparum*				43 (including co-infections)	
14	Demissie et al., 2009	Ethiopia (2006)	Cross-sectional study	370 suspected malaria patients, 120 malaria cases (77 *P. falciparum*, 33 *P. vivax*)	44	10.72 (9.8–11.6) or 10.7 ± 0.56		8/44 severe anemia, 12/44 moderate anemia, 3/44 mild anemia, 21/44 non-anamia	76 malaria without hookworm	11.7 (11.2–11.3) or 11.7 ± 0.38		20/102	141 (including co-infections)	12.77 (19)
15	Egwunyenga et al., 2001	Nigeria (1997–1998)	Cross-sectional study	2104 pregnant women, 816 (762 *P. falciparum*, 54 *P. malariae*)	116				422	Bauchi (9.7 ± 1.5, 10.1 ± 1.2), Jos (10.3 ± 1.4, 10.5 ± 1.2), Eku (8.9 ± 1.9, 10.5 ± 1.6)			NA	
16	Ekejindu et al., 2011	Nigeria	Cross-sectional study	100 pregnant women and 100 non-pregnant women, 152 malaria cases	19 (pregnant 13, non-pregnant 6)				Pregnant: 81 *P. falciparum* Non-pregnant: 52 malaria cases				Pregnant: 17 Non-pregnant: 9	
17	Getaneh et al., 2020	Ethiopia (2019)	Cross-sectional study	2675 malaria-suspected patients, 512 malaria cases and 134 were included	54/134, 15,063:64 ± 14,628:96 Light infection 8392.5, moderate infection 10,244.6, Heavy infection 26,230				67/134, 7543.12 ± 8541.292		< 15 (9), 15–45 (50), > 45 (8), 32/67		NA	
18	Hailu et al., 2018	Ethiopia (2016)	Cross-sectional study	333 febrile school age children, 143 (137 *P. falciparum*, 6 *P. vivax*)	18 (15 *P. falciparum*, 3 *P. vivax*)	*P. falciparum* 9.2 (8.72–9.68) or 9.2 ± 0.34, 15		16/18	112 *P. falciparum*	*P. falciparum* 10.79 (10.53–11.04) or 10.8 ± 0.25, 112			37 (including co-infections)	11.33 ± 1.05 (159)
19	Hillier et al., 2008	Uganda (2000–2001)	Cross-sectional study	2507 pregnant women, 268/2459 *P. falciparum*	138 *P. falciparum*				118/1278 *P. falciparum*				1,112 (including co-infections)	
20	Humphries et al., 2013	Ghana (2010)	Cross-sectional study	286 school children, 210/249 (205 *P. falciparum*)	100				110				109 (including co-infections)	
21	Hurlimann et al., 2019	Coˆte d’Ivoire (2011–2013)	Cross-sectional study	6245 participants, 4530 malaria cases 706 adults, 322 malaria cases 601 school-aged children/adolescents, 507 malaria cases 4938 national survey school-aged children/adolescents, 3701 malaria cases	2979 Anemia/non-anemia 199/427		1646/2979/ mean 10.1	928/2,979	1551 malaria cases without hookworm Anemia/non-anemia 568/1172				1,229/4,208 706 adults, 222 hookworm 601 school-aged children/adolescents, 156 hookworm 4,938 school-aged children/adolescents, 851 hookworm Anemia/non-anemia 35/84	
22	Kabatereine et al., 2011	Uganda (2009–2010)	Cross-sectional study	5016 school-age children, 1724/3712 malaria cases	289				1231				674 (including co-infections)	
23	Kepha et al., 2015	Kenya (2013)	Cross-sectional study	5471 school-age children, 2541 *P. falciparum*	494 *P. falciparum*				1757 *P. falciparum*				169/922 (18.3%)	
24	Kinung’hi et al., 2014	Tanzania (2006)	Cross-sectional study	1546 children, 460 *P. falciparum*	20 *P. falciparum*	12.6	35		184 *P. falciparum*	12.2		40	245 (including co-infections)	12.6 ± 0.75 (467)
25	Mazigo et al., 2010	Tanzania (2009)	Cross-sectional study	400 school children, 57 (54 *P. falciparum*, 3 *P. ovale*)	9 *P. falciparum*		11–13 (3), 14–16 (6) Male 1/6		47 *P. falciparum* Pure *P. falciparum*				152 (including co-infections)	
26	Mboera et al., 2011	Tanzania (2005)	Cross-sectional study	578 school children, 362 *P. falciparum*	31 *P. falciparum*, 493.1		Male 20/31	25	204 *P. falciparum*, 261.2			70%	59	
27	Melo et al., 2010	Brazil (2008)	Cross-sectional study	236 Children, 83/216 malaria cases (24 *P. falciparum*, 56 *P. vivax*, 3 mixed infection)	11 *P. vivax*, 1,805	11.7	5–11 (8), 12–14 (3), 5/11		43 *P. vivax*, 2874.2	12	5–11 (34), 12–14 (9), 21/22		NA	NA
28	Muller et al., 2011	Coˆte d’Ivoire (2009–2010)	Cross-sectional study	204 school children, 111 malaria cases	1				11				21 (including co-infections)	
29	Mulu et al., 2013	Ethiopia (2006)	Cross-sectional study	463 children, 230 malaria cases (134 *P. falciparum*, 81 *P. vivax*, 15 mixed infection)	23				76				43 (including co-infections)	
30	Nkuo-Akenji et al., 2006	Cameroon (2004)	Cross-sectional study	425 children, 170 *P. falciparum*	1 *P. falciparum*	PCV (12)	6–10 (1), 0/1	Severe anemia (1)	169 *P. falciparum*	PCV (25.9 ± 4.86)	≤ 5 (43), 6–10 (13), 11–14 (4), 31/60	Mild anemia (53), moderate anemia (6), severe anemia (1)	1	
31	Oboth et al., 2019	Uganda (2017–2018)	Cross-sectional study	476 children, 262 *P. falciparum*	1 *P. falciparum*				234 *P. falciparum*				1	
32	Ojurongbe et al., 2011	Nigeria (2009)	Cross-sectional study	117 school pupils, 30 *P. falciparum*	2 *P. falciparum*				23 *P. falciparum*				4	
33	Ojurongbe et al., 2018	Nigeria (2012–2013)	Cross-sectional study	200 pregnant women, 59 *P. falciparum*	1 *P. falciparum*				49 *P. falciparum*				3	
34	Pullan et al., 2010	Uganda (2008)	Cross-sectional study	1,770 participants, 687 malaria cases	274, < 5 (54), 5–15 (131), ≥ 16 (89)	< 5 (10), 5–15 (11.8), ≥ 16 (12.6)	< 5 (51), 5–15 (131), ≥ 16 (49)		< 5 (169), 5–15 (381), ≥ 16 (139)				694 < 5 (93), 5–15 (204), ≥ 16 (397)	
35	Righetti et al., 2012	Coˆte d’Ivoire (2010)	Cross-sectional study	732 participants, 425 *P. falciparum*	46, 6 years old (1), 138.6 (23.2–840.7) 7 years old, 678.8 (227.0–2017.3) 8 years old, 825.5 (480.5–1,417.8)	6 years old (11 ± 1.0), 7 years old (11.9 ± 1.0), 8 year old (11.9 ± 1.0)			6 years old, 1141.3 (495.6–2626.4) 7 years old, 490.9 (214.4–1122.1) 8 years old, 280.5 (61.2–1,272.7)	6 years old (11.3 ± 1.0), 7 years old (11.4 ± 1.0), 8 year old (11 ± 1.1)			70, 6 years old (1), 7 years old (44), 8 years old (25)	
36	Salim et al., 2015	Tanzania (2011–2012)	Cross-sectional study	1033 children, 130 malaria cases	14				80				60	
37	Shittu et al., 2017	Nigeria (2014–2015)	Cross-sectional study	700 voluntaries donors, 372 malaria cases	168, 7346.19 ± 221.701				187, 3676.91 ± 157.077 (372)				291 (including co-infections)	

Most of the included studies were conducted in school-age children (19/37, 51.4%)^9,20,24,29,30,35–45,49,52,53^, pregnant women (7/37, 18.9%)^25,28,32–34,46,51^, residents in the community (6/37, 16.2%)^10,11,26,27,47,48^, and acute febrile patients (4/37, 10.8%)^8,12,13,31^, while 1 study was conducted in voluntary donors^50^. Of the 37 studies included in the present analysis, 22,191 participants were confirmed to have malaria infection. Among those malarial patients, 6096 cases were patients with *Plasmodium* spp. and hookworm co-infection.

### Prevalence of *Plasmodium* spp. and hookworm co-infection

**Figure 2:**
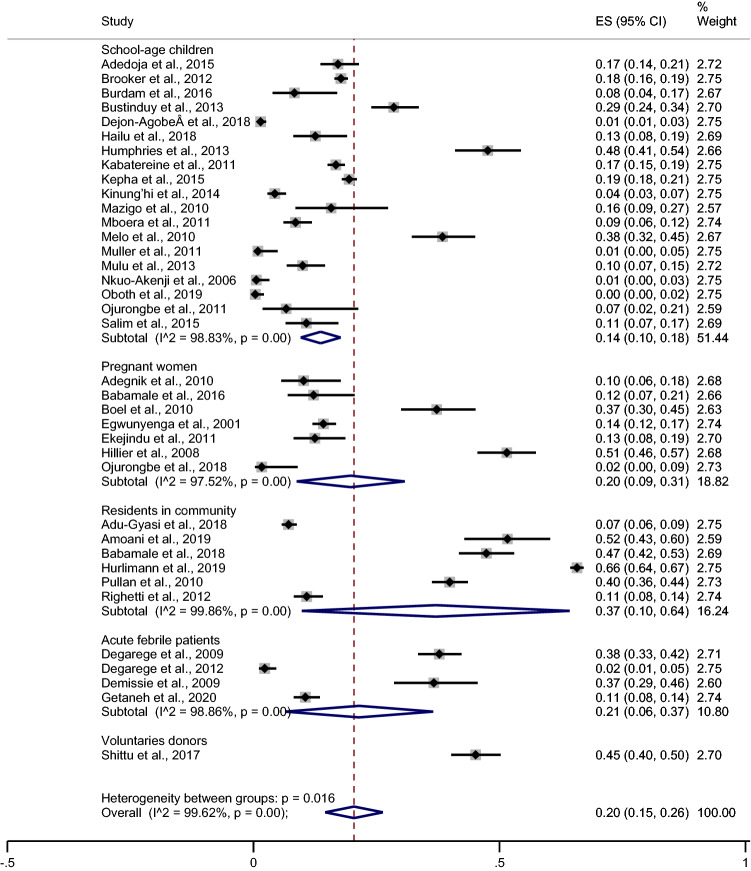
Pooled prevalence of *Plasmodium* spp. and hookworm co-infection stratified by participants. *ES* prevalence estimates (× 100), *CI* confidence interval.

The pooled prevalence of *Plasmodium* spp. and hookworm co-infection was estimated from 37 studies. The result demonstrated the pooled prevalence of *Plasmodium* spp., and hookworm co-infection was 20% (95% CI 15–26%, I^2^ 99.6%). The meta-regression analysis was performed to identify the source (s) of heterogeneity of the prevalence. The meta-regression analysis using participant types as covariates showed that differences in participant type were not the source of heterogeneity of the pooled prevalence of *Plasmodium* spp. and hookworm co-infection (p > 0.05). A subgroup analysis of participants demonstrated that the pooled prevalence of co-infection was highest in residents in the community (37%, 95% CI 10–64%, I^2^ 99.9%), acute febrile patients (21%, 95% CI 6–37%, I^2^ 98.9%), pregnant women (20%, 95% CI 9–31%, I^2^ 97.5%) and school-age children (14%, 95% CI 10–18%, I^2^ 98.8%). One study conducted on voluntary donors demonstrated 45% (95% CI 40–50) co-infection^50^. The results of the individual study demonstrated that the prevalence of *Plasmodium* spp. and hookworm co-infection was highest in a study by Amoani et al. (52%)^27^, Hillier et al. (51%)^34^, Humphries et al. (48%)^35^ and Babamale et al. (47%)^11^, respectively (Fig. 2).

**Figure 3:**
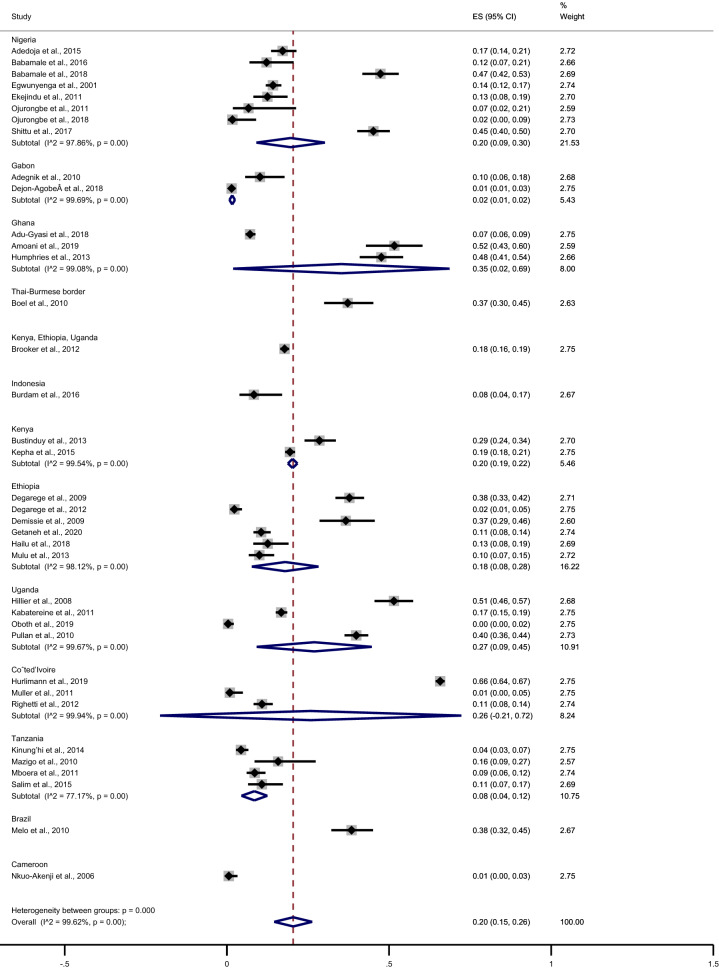
Pooled prevalence of *Plasmodium* spp. and hookworm co-infection stratified by countries. *ES* prevalence estimates (× 100), *CI* confidence interval.

The meta-regression analysis using countries as covariates showed that differences in participant type were a source of heterogeneity of the pooled prevalence of *Plasmodium* spp. and hookworm co-infection (p < 0.05). Further subgroup analysis of countries yielded the following results: Nigeria (20%, 95% CI 9–30%, I^2^ 97.9%), Gabon (2%, 95% CI 1–2%, I^2^ 99.7%), Ghana (35%, 95% CI 2–69%, I^2^ 99.1%), Kenya (20%, 95% CI 19–22%, I^2^ 99.5%), Ethiopia (18%, 95% CI 8–28%, I^2^ 98.1%), Uganda (27%, 95% CI 9–45% I^2^ 99.7%), Coˆted’Ivoire (26%, 95% CI 0–72%, I^2^ 99.9%), Tanzania (8%, 95% CI 4–12%, I^2^ 77.2%) (Fig. 3).

### Quality of the included studies

Ten studies^8,9,11,13,24,28,38,40,50,53^ were rated as high-quality studies, since they reported the outcomes of interest, whereas the rest of the studies were rated as moderate quality, since they reported only the data of co-infection cases but not data on haemoglobin and malaria parasite density (Supplementary Table S2).

### POR of *Plasmodium* spp. and hookworm co-infection

**Figure 4:**
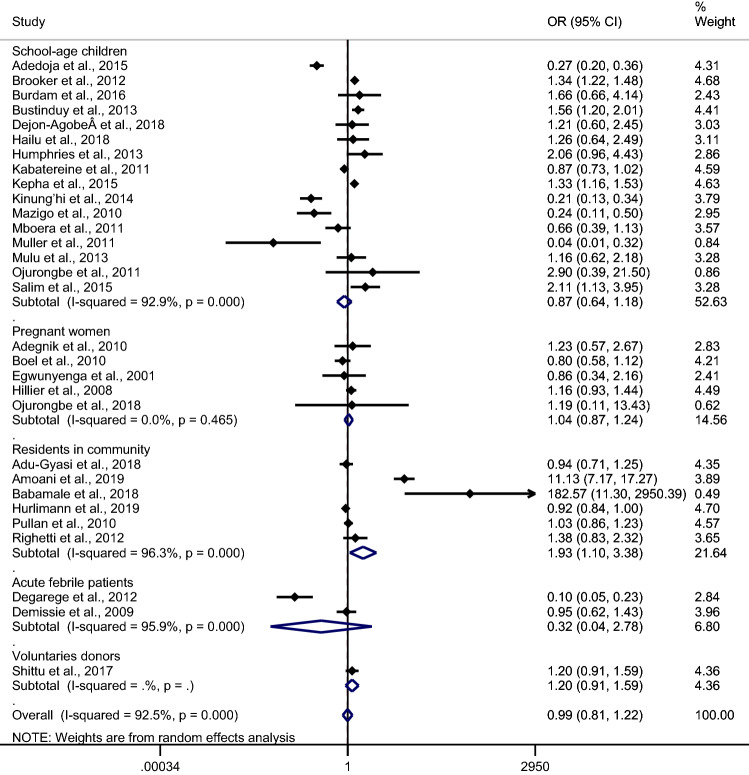
The prevalence odds ratio of *Plasmodium* spp. and hookworm co-infection. *POR* prevalence odds ratio, *CI* confidence interval.

The number of hookworm infections in malaria-positive patients and malaria-negative patients were analysed to determine if co-infection occurred by chance. Overall, the meta-analysis of 30 studies demonstrated that co-infection occurred by chance (*p* 0.94, OR 0.99, 95% CI 0.81–1.22, I^2^ 92.5%). A subgroup analysis of participants demonstrated that studies conducted among residents in communities increased the pooled POR of co-infection (*p* 0.023, OR 1.93, 95% CI 1.10–3.38, I^2^ 96.3%). Three studies by Salim et al.^49^, Amoani et al.^27^ and Babamale et al.^11^ demonstrated increased POR for co-infection, while 5 studies by Adedoja et al.^24^, Kinung’hi et al.^38^, Mazigo et al.^39^, Muller et al.^41^ and Degarege et al.^31^ demonstrated decreased POR of co-infection (Fig. 4).

### *Plasmodium* spp. and hookworm co-infection and malaria parasite density

**Figure 5:**
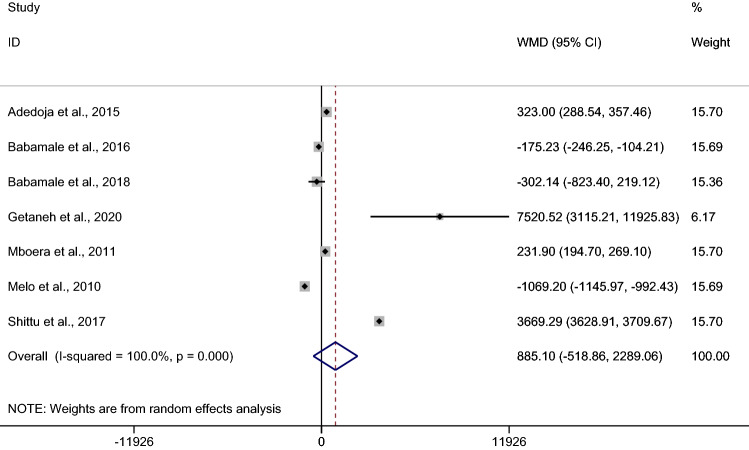
Mean malaria parasite density between patients with *Plasmodium* spp. and hookworm co-infection and *Plasmodium* mono-infection. *WMD* weighted mean difference, *CI* confidence interval.

The pooled MD of malaria parasite density between patients with co-infection (478 cases) and *Plasmodium* mono-infection (920 cases) was estimated from 7 studies^11,13,24,28,40,50,53^. The results demonstrated no difference in the mean malaria parasite density between patients with co-infection and *Plasmodium* mono-infection when a random effect model was used (*p* 0.22, MD 885.1, 95% CI − 518.9–2289.1, I^2^ 100%). Four studies^13,24,40,50^ demonstrated a higher mean of malaria parasite density in co-infection than in *Plasmodium* mono-infection, while two studies^28,53^ demonstrated a lower mean of malaria parasite density in co-infection than in *Plasmodium* mono-infection (Fig. 5). When excluding the study by Getaneh et al.^13^, which reported a high mean parasite density in patients with co-infection (outliner), no difference in the mean malaria parasite density was found between patients with co-infection and *Plasmodium* mono-infection (*p* 0.54, MD 449, 95% CI − 1000–1898.2, I^2^ 100%) (Supplementary Fig. 1).

### *Plasmodium* spp. and hookworm co-infection and haemoglobin level

**Figure 6:**
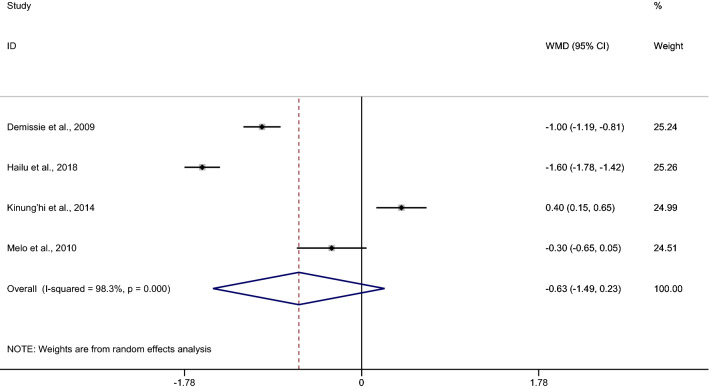
Mean haemoglobin levels between patients with *Plasmodium* spp. and hookworm co-infection and *Plasmodium* mono-infection. *WMD* weighted mean difference, *CI* confidence interval.

**Figure 7:**
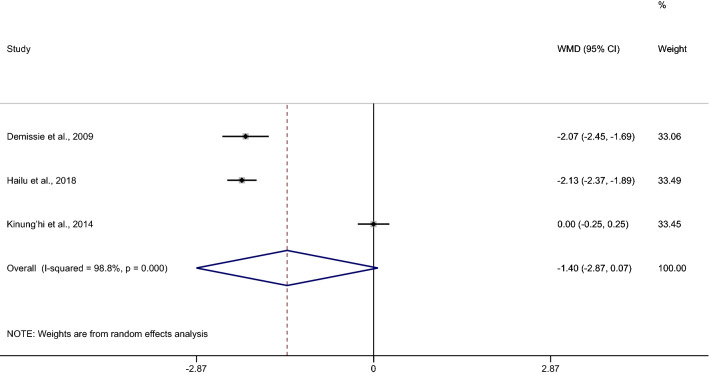
Mean hemoglobin levels between patients with *Plasmodium* spp. and hookworm co-infection and patients without any infection. *WMD* weighted mean difference, *CI* confidence interval.

The pooled MD of haemoglobin between patients with co-infection (90 cases) and *Plasmodium* mono-infection (415 cases) was estimated from four studies^8,9,38,53^. The results demonstrated no difference in the mean haemoglobin level between patients with co-infection and *Plasmodium* mono-infection when a random effect model was used (*p* 0.152, MD − 0.63, 95% CI − 1.49–0.23, I^2^ 98.3%). Two studies^8,9^ demonstrated a lower mean haemoglobin level in co-infection than in *Plasmodium* mono-infection, whereas one study^38^ demonstrated a higher mean haemoglobin level in co-infection than in *Plasmodium* mono-infection (Fig. 6). The pooled MD of haemoglobin between patients with co-infection (79 cases) and without any infection (645 cases) was estimated from three studies^8,9,38^. The results demonstrated no difference in the mean haemoglobin level between patients with co-infection and without any infection when a random effects model was used (*p* 0.062, MD − 1.4, 95% CI − 2.87 to 0.07, I^2^ 98.8%) (Fig. 7).

### Publication bias

**Figure 8:**
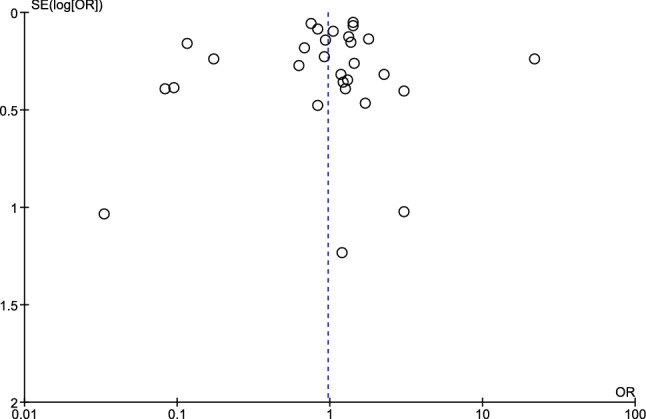
The funnel plot demonstrated the distribution of prevalence odds ratio of *Plasmodium* spp. and hookworm co-infection (ES) and standard error (SE) of the ES. *ES* prevalence estimate, *SE* standard error.

Publication bias was assessed using the funnel plot demonstrating the effect size (pooled OR) and selogES from 30 studies (Fig. 8). Egger’s test demonstrated that no small-study effect was found (p 0.128, coefficiency 6.77, standard error 4.31). Visualisation of the funnel plot and the result of Egger’s test demonstrated asymmetrical distribution of the funnel plot, and no small-study effect was found among the included studies, respectively.

## Discussion

The effects of *Plasmodium* spp. co-infection with other diseases can cause serious clinical outcomes^54–58^. Co-infection of *Plasmodium* spp. and hookworm occurs in SSA; however, the mechanism and impact of the co-infection on the severity of malaria disease remain unknown. The present study demonstrated that 91.9% of the included studies reported *Plasmodium* spp. and hookworm co-infection in Africa, including Nigeria, Ethiopia, Tanzania, Uganda, Coˆted’Ivoire, Ghana, Kenya, Gabon and Cameroon. Some studies reported *Plasmodium* spp. and hookworm in the Thai-Burmese border^51^, Indonesia^52^ and Brazil^53^. In Africa, the highest pooled prevalence of co-infection was reported in Ghana (35%), Uganda (27%), Coˆted’Ivoire (26%), Nigeria (20%), Kenya (20%) and Ethiopia (18%), while a low pooled prevalence of co-infection was reported in Tanzania (8%), Gabon (2%) and Cameroon (1%). Based on those results, the difference in pooled prevalence was the difference in geographic area, which introduce different environmental factors, or the occurrence of malaria or hookworm per population in each area. The difference in the prevalence of co-infection suggested a difference in the geographic region, which have different environmental factors for hookworm infection. Previous studies showed that hookworm prevalence in SSA (14–43%) varied^9,59,60^. For example, previous studies demonstrated that hookworm is endemic in some communities of Ghana, with a high prevalence rate up to 59%^35,61^, and was correlated with a high prevalence of co-infection as estimated in the present study. In malaria-endemic areas, it is plausible that hookworm infection might suppress the inflammatory responses caused by *Plasmodium* spp., thereby reducing the risk of severe malaria^62^. Another contributing factor for a difference in the pooled prevalence of co-infection might be the participants investigated. The subgroup analysis of participants demonstrated that the pooled prevalence of co-infection was highest in residents in the community (37%), acute febrile patients (21%), pregnant women (20%), school-age children (14%) and voluntary donors (45%). Curiously, the higher prevalence of *Plasmodium* spp. and hookworm co-infection was more common among residents in the community than in school-age children. The present pooled analysis observed a high rate of co-infection in pregnant women (20%). Among pregnant women, the highest rate of co-infection was demonstrated in the study by Hillier et al. (51%)^34^ and Boel et al. (37%)^51^. A possible explanation for the high rate of co-infection in these participants may be attributed to the impairment of immunity during pregnancy^63^. A study demonstrated that that pregnant women are more susceptible to *Plasmodium* spp. and hookworm infections in their first pregnancy, which might cause nutrient deficiency in subjects, which would lead to poor pregnancy outcomes^33^.

The co-infection of *Plasmodium* spp. and hookworm might occur by chance. The pooled analysis of this study suggested that underlying infection by hookworms may not increase the chance of being infected with malaria. Nevertheless, the meta-analysis for each subgroup of participants demonstrated that hookworm infections of people within communities increased the risk of malaria infection. Previous studies showed that rural communities are associated with poverty, poor sanitation and personal hygiene, and in turn, are related to soil-transmitted-helminth infection, including hookworm infection^64–66^. Four studies conducted in school-age children demonstrated that hookworm infection decreased susceptibility to malaria infection^24,38,39,41^. As for the study in school-age children, another study conducted in acutely febrile patients demonstrated that hookworm infection decreased susceptibility to malaria infection^31^. On the contrary, previous studies conducted in school-age children and residents in communities demonstrated that hookworm infection increased susceptibility to malaria infection^11,27,29,37,49^. The protective or risk factors for malaria infection by hookworm infection were poorly understood. A previous study suggested that immunological interactions, micro-geographical variation, socioeconomic variables and spatial distribution of environmental conditions favour the transmission of multiple species^25,34,62,67^. Moreover, a previous study revealed considerable variation in the probability of *Plasmodium* and hookworm co-infection by geographic location, and the co-infection occurred frequently in zones where the prevalence of hookworm and *P. falciparum* infection were highest^34^. Regarding STH co-infection with *Plasmodium* spp., previous studies demonstrated that co-infection was more common among boys, less common with increasing age and highest among children from poor households^29,48,68,69^. Therefore, the risk of *Plasmodium* spp. and hookworm might be associated with access to sanitation and clean water, recent deworming and living in urban settings^69^, as in these areas, children are exposed to open defecation grounds, which is a major source of hookworm transmission infection^70^.

The present meta-analysis demonstrated that there was no difference in malaria parasite density among patients with co-infection when compared to patients with *Plasmodium* spp. mono-infection. Only two studies demonstrated that a higher hookworm intensity was positively correlated with higher malaria parasite density^13,50^. Previous studies demonstrated that a higher hookworm intensity was positively correlated with a higher malaria parasite density, whereas it was negatively correlated with a lower malaria parasite density when malaria was co-infected with *A. lumbricoides*^12,13^. The possible association of hookworm with protection from severe malaria was that infection of helminth modulates inflammatory factors and immunoglobulin E-induced nitric oxide (NO) production and is related to reduced parasite sequestration, which protects against severe malaria^71,72^. In addition, helminth may decrease cytophilic IgG1 and IgG3 antibodies, which protect the host from malaria disease. Moreover, helminth infection can increase the non-cytophilic IgG2, IgG4 and IgM antibodies, thereby accelerating the severity of malaria^73^.

Anaemia caused by malaria and hookworm is attributable to a combination of chronic blood loss, haemolysis, and haemopoietic suppression^52^. In addition, children with asymptomatic *Plasmodium* infection demonstrated impaired intestinal iron absorption, which may play an important role in the development of anaemia^74^. Previous studies demonstrated that hookworm infection is more prevalent in older children than in young children, and is associated with chronic intestinal blood loss^62,75^. In areas where co-infection was low, co-infection was related to anaemia and its effect on the child's health and development^62^. The mechanisms by which *Plasmodium* spp. causes anaemia involve decreased red blood cell production by bone marrow suppression and increased red blood cell destruction through rupturing, phagocytosis and hypersplenism^76,77^, while hookworm infection contributes to anaemia through chronic blood loss in the intestine^78^. The present study found no association between co-infection and malaria parasite density or haemoglobin level among the included studies. A possible explanation is that the impact of co-infection on these parameters might be due to an increase in the number of intestinal helminths species than only hookworm co-infection^12,71^. In addition, when compared to other intestinal helminth infections, the mean *Plasmodium* density in co-infected individuals with hookworm was lower than in malarial patients co-infected with *A. lumbricoides* alone, *S. mansoni* alone or *T. trichiura* alone^31^. Another explanation for the difference in contradicting reports about parasite density among co-infected patients is the variation among the included studies, such as the difference in participants, study design, methodology, level of parasite endemicity and local climate. These variations impact the differences in *Plasmodium* spp. and hookworm interactions and influence the heterogeneity among the includes studies. Another explanation suggested by a previous study was that hookworm infection was not associated with anaemia if low infection intensities were detected in the studied population^38,79^. The non-impact of *Plasmodium* spp. and hookworm co-infection on anaemia in the present study suggested that the anaemia was most likely due to dietary deficiency. Therefore, more studies are needed to explore the impact of co-infection on anaemia.

This study has several limitations. First, several important sources of databases, such as ScienceDirect and OVID, were not included in the search. Therefore, some relevant studies may have been missed from the search. Second, the source of heterogeneity across the included studies in the pooled prevalence analysis cannot be explored due to the limited data on the included studies. Therefore, a pooled analysis needed to be interpreted with caution. Third, the number of included studies that reported the mean or median haemoglobin and mean malarial parasite density was limited, which caused imprecision in the estimate for a pooled analysis of the mean haemoglobin and mean parasite density between patients with *Plasmodium* spp. and hookworm infection. Third, only studies published in English were included. For this reason, studies in Latin America are absent in this review, although this region has high malaria and hookworm endemicity.

In conclusion, co-infection of *Plasmodium* spp. and hookworm was common and it most likely occurred by chance. The meta-analysis demonstrated no difference in the malaria parasite density and haemoglobin level in patients with co-infection compared to *Plasmodium* monoinfection. However, these results were based on the limited number of studies included for meta-analysis. Therefore, for a more comprehensive review, further meta-analysis studies should include non-English literature or case–control studies. Additionally, further studies are needed to investigate the mechanism of hookworm infection on malaria severity. Finally, the detection of hookworm infections among patients with malaria in endemic areas of both diseases is recommended to prevent severe malaria.

## Supplementary Information

Supplementary Information 1.

Supplementary Information 2.

Supplementary Figure 1.

Supplementary Table S1.

Supplementary Table S2.

## Data Availability

All data supporting the findings of this study are available within the article and its supplementary files.
