# Supplementary figures and images for: Prevalence and effect of Plasmodium spp. and hookworm co-infection on malaria parasite density and haemoglobin level: a meta-analysis
Source: Sci Rep. 2022 Apr 27;12:6864. doi: 10.1038/s41598-022-10569-2 (PMC9046215; doi:10.1038/s41598-022-10569-2)

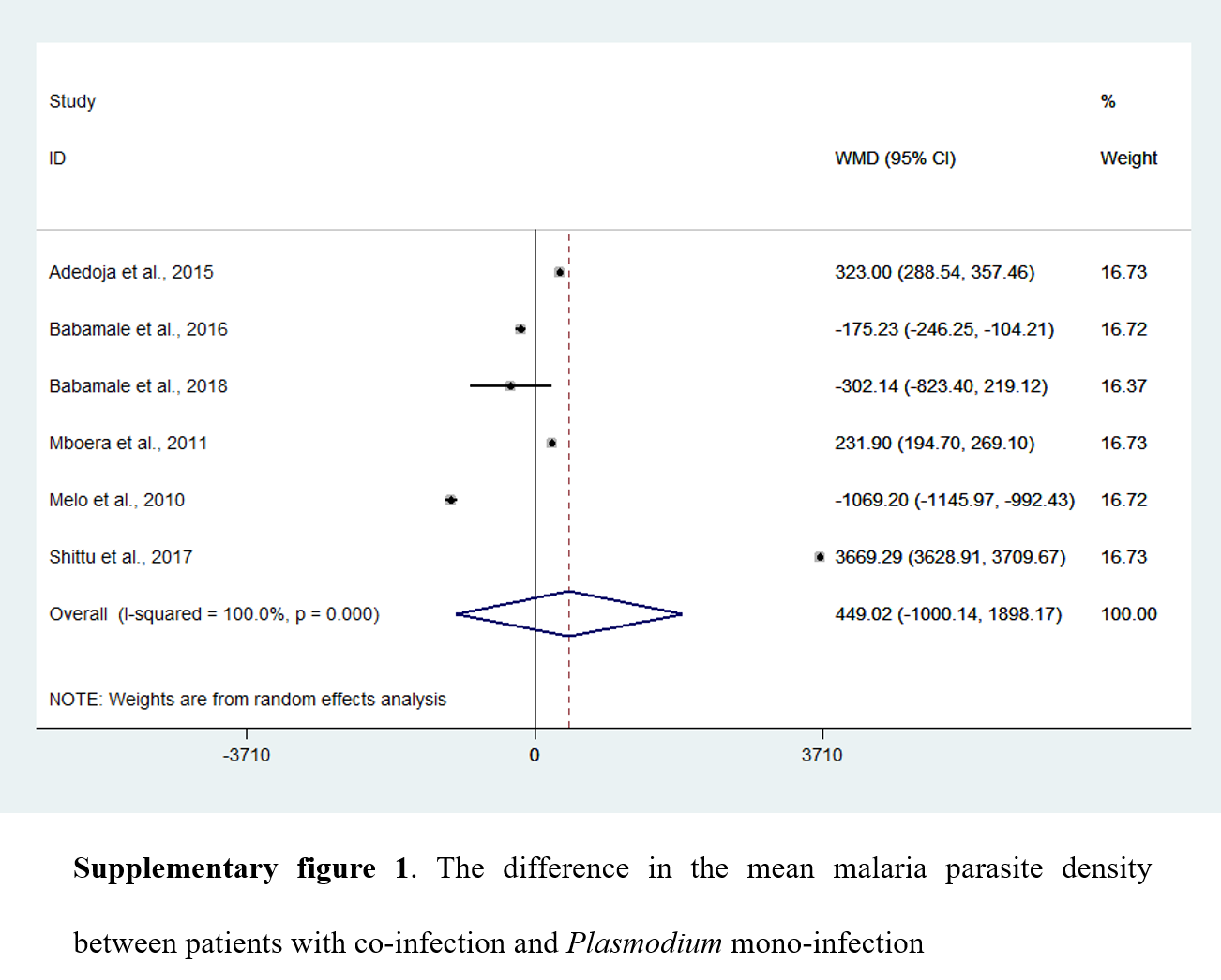

Supplement: Supplementary file 3 — Supplementary Figure 1. [file 41598_2022_10569_MOESM3_ESM.png]
