# Supplementary material for: Prevalence and effect of Plasmodium spp. and hookworm co-infection on malaria parasite density and haemoglobin level: a meta-analysis
Source: Sci Rep. 2022 Apr 27;12:6864. doi: 10.1038/s41598-022-10569-2 (PMC9046215; doi:10.1038/s41598-022-10569-2)
Supplement: Supplementary file 4 — Supplementary Table S1. [file 41598_2022_10569_MOESM4_ESM.docx]

**Prevalence and effect of *Plasmodium* spp. and hookworm co-infection on malaria parasite density and haemoglobin level: A meta-analysis**

Aongart Mahittikorn ^1^, Frederick Ramirez Masangkay ^2^, Giovanni De Jesus Milanez ^2^, Saruda Kuraeiad ^3^, Manas Kotepui ^3*^

^1^ Department of Protozoology, Faculty of Tropical Medicine, Mahidol University, Bangkok, Thailand

^2^ Department of Medical Technology, Institute of Arts and Sciences, Far Eastern University-Manila, Manila, Philippines

^3^ Medical Technology, School of Allied Health Sciences, Walailak University, Tha Sala, Nakhon Si Thammarat, Thailand

Authors’ Email Addresses:

**^*^Corresponding Author**: Manas Kotepui; manaskote@gmail.com

Aongart Mahittikorn; aongart.mah@mahidol.ac.th

Frederick Ramirez Masangkay; frederick_masangkay2002@yahoo.com

Kwuntida Uthaisar Kotepui; kwuntida.ut@wu.ac.th

Giovanni De Jesus Milanez; gmilanez@feu.edu.ph

**S1 Table. Search term**

| **Databases** | **Search terms** | **Search date** |
| --- | --- | --- |
| MEDLINE | (malaria OR Plasmodium) AND (hookworm OR Ascaris OR Necator) | 24 December 2020 |
| Scopus | (malaria OR Plasmodium) AND (hookworm OR Ascaris OR Necator)  Search option: Article title, abstract, keyword | 24 December 2020 |
| ISI Web of Science | (malaria OR Plasmodium) AND (hookworm OR Ascaris OR Necator)  Search option: All fields | 24 December 2020 |
