# Supplementary material for: Prevalence and effect of Plasmodium spp. and hookworm co-infection on malaria parasite density and haemoglobin level: a meta-analysis
Source: Sci Rep. 2022 Apr 27;12:6864. doi: 10.1038/s41598-022-10569-2 (PMC9046215; doi:10.1038/s41598-022-10569-2)
Supplement: Supplementary file 5 — Supplementary Table S2. [file 41598_2022_10569_MOESM5_ESM.docx]

**Prevalence and effect of *Plasmodium* spp. and hookworm co-infection on malaria parasite density and haemoglobin level: A meta-analysis**

Aongart Mahittikorn ^1^, Frederick Ramirez Masangkay ^2^, Giovanni De Jesus Milanez ^2^, Saruda Kuraeiad ^3^, Manas Kotepui ^3*^

^1^ Department of Protozoology, Faculty of Tropical Medicine, Mahidol University, Bangkok, Thailand

^2^ Department of Medical Technology, Institute of Arts and Sciences, Far Eastern University-Manila, Manila, Philippines

^3^ Medical Technology, School of Allied Health Sciences, Walailak University, Tha Sala, Nakhon Si Thammarat, Thailand

Authors’ Email Addresses:

**^*^Corresponding Author**: Manas Kotepui; manaskote@gmail.com

Aongart Mahittikorn; aongart.mah@mahidol.ac.th

Frederick Ramirez Masangkay; frederick_masangkay2002@yahoo.com

Kwuntida Uthaisar Kotepui; kwuntida.ut@wu.ac.th

Giovanni De Jesus Milanez; gmilanez@feu.edu.ph

**Table S2.** Quality of the included studies

| **No.** | **Author, year, reference number** | **Selection** | | | | **Compatibility** | **Exposure** | | | **Total score (7)** | **Rating (High, moderate, low quality)** |
| --- | --- | --- | --- | --- | --- | --- | --- | --- | --- | --- | --- |
|  |  | **Is the Case Definition Adequate?** | **Representativeness of the Cases** | **Selection of Controls** | **Definition of Controls** |  | **Ascertainment of Exposure** | **Same method of ascertainment for cases and controls** | **Non-Response Rate** |  |  |
| 1. | Adedoja et al., 2015 | 🟑 | 🟑 | 🟑 | 🟑 | 🟑 | 🟑 | 🟑 | NA | 7/7 | High quality |
| 2 | Adegnik et al., 2010 | 🟑 | 🟑 |  |  | NA | NA | NA | NA | 2/4 | Moderate quality |
| 3. | Adu-Gyasi et al., 2018 | 🟑 | 🟑 |  |  | NA | NA | NA | NA | 2/4 | Moderate quality |
| 4. | Amoani et al., 2019 | 🟑 | 🟑 |  |  | NA | NA | NA | NA | 2/4 | Moderate quality |
| 5. | Babamale et al., 2016 | 🟑 | 🟑 | 🟑 | 🟑 | 🟑 | 🟑 | 🟑 | NA | 7/7 | High quality |
| 6. | Babamale et al., 2018 | 🟑 | 🟑 | 🟑 | 🟑 | 🟑 | 🟑 | 🟑 | NA | 7/7 | High quality |
| 7. | Boel et al., 2010 | 🟑 | 🟑 |  |  | NA | NA | NA | NA | 2/4 | Moderate quality |
| 8. | Brooker et al., 2012 | 🟑 | 🟑 |  |  | NA | NA | NA | NA | 2/4 | Moderate quality |
| 9. | Burdam et al., 2016 | 🟑 | 🟑 |  |  | NA | NA | NA | NA | 2/4 | Moderate quality |
| 10. | Bustinduy et al., 2013 | 🟑 | 🟑 |  |  | NA | NA | NA | NA | 2/4 | Moderate quality |
| 11. | Degarege et al., 2009 | 🟑 | 🟑 |  |  | NA | NA | NA | NA | 2/4 | Moderate quality |
| 12. | Degarege et al., 2012 | 🟑 | 🟑 |  |  | NA | NA | NA | NA | 2/4 | Moderate quality |
| 13. | Dejon-AgobeÂ et al., 2018 | 🟑 | 🟑 |  |  | NA | NA | NA | NA | 2/4 | Moderate quality |
| 14. | Demissie et al., 2009 | 🟑 | 🟑 | 🟑 | 🟑 | 🟑 | 🟑 | 🟑 | NA | 7/7 | High quality |
| 15. | Egwunyenga et al., 2001 | 🟑 | 🟑 |  |  | NA | NA | NA | NA | 2/4 | Moderate quality |
| 16. | Ekejindu et al., 2011 | 🟑 | 🟑 |  |  | NA | NA | NA | NA | 2/4 | Moderate quality |
| 17. | Getaneh et al., 2020 | 🟑 | 🟑 | 🟑 | 🟑 | 🟑 | 🟑 | 🟑 | NA | 7/7 | High quality |
| 18 | Hailu et al., 2018 | 🟑 | 🟑 | 🟑 | 🟑 | 🟑 | 🟑 | 🟑 | NA | 7/7 | High quality |
| 19. | Hillier et al., 2008 | 🟑 | 🟑 |  |  | NA | NA | NA | NA | 2/4 | Moderate quality |
| 20. | Humphries et al., 2013 | 🟑 | 🟑 |  |  | NA | NA | NA | NA | 2/4 | Moderate quality |
| 21. | Hurlimann et al., 2019 | 🟑 | 🟑 |  |  | NA | NA | NA | NA | 2/4 | Moderate quality |
| 22. | Kabatereine et al., 2011 | 🟑 | 🟑 |  |  | NA | NA | NA | NA | 2/4 | Moderate quality |
| 23. | Kepha et al., 2015 | 🟑 | 🟑 |  |  | NA | NA | NA | NA | 2/4 | Moderate quality |
| 24. | Kinung’hi et al., 2014 | 🟑 | 🟑 | 🟑 | 🟑 | 🟑 | 🟑 | 🟑 | NA | 7/7 | High quality |
| 25. | Mazigo et al., 2010 | 🟑 | 🟑 |  |  | NA | NA | NA | NA | 2/4 | Moderate quality |
| 26. | Mboera et al., 2011 | 🟑 | 🟑 | 🟑 | 🟑 | 🟑 | 🟑 | 🟑 | NA | 7/7 | High quality |
| 27. | Melo et al., 2010 | 🟑 | 🟑 | 🟑 | 🟑 | 🟑 | 🟑 | 🟑 | NA | 7/7 | High quality |
| 28. | Muller et al., 2011 | 🟑 | 🟑 |  |  | NA | NA | NA | NA | 2/4 | Moderate quality |
| 29. | Mulu et al., 2013 | 🟑 | 🟑 |  |  | NA | NA | NA | NA | 2/4 | Moderate quality |
| 30. | Nkuo-Akenji et al., 2006 | 🟑 | 🟑 |  |  | NA | NA | NA | NA | 2/4 | Moderate quality |
| 31. | Oboth et al., 2019 | 🟑 | 🟑 |  |  | NA | NA | NA | NA | 2/4 | Moderate quality |
| 32. | Ojurongbe et al., 2011 | 🟑 | 🟑 |  |  | NA | NA | NA | NA | 2/4 | Moderate quality |
| 33. | Ojurongbe et al., 2018 | 🟑 | 🟑 |  |  | NA | NA | NA | NA | 2/4 | Moderate quality |
| 34. | Pullan et al., 2010 | 🟑 | 🟑 |  |  | NA | NA | NA | NA | 2/4 | Moderate quality |
| 35. | Righetti et al., 2012 | 🟑 | 🟑 |  |  | NA | NA | NA | NA | 2/4 | Moderate quality |
| 36. | Salim et al., 2015 | 🟑 | 🟑 |  |  | NA | NA | NA | NA | 2/4 | Moderate quality |
| 37. | Shittu et al., 2017 | 🟑 | 🟑 | 🟑 | 🟑 | 🟑 | 🟑 | 🟑 | NA | 7/7 | High quality |

🟑 A star rating
